# RGD Peptide Modified Erythrocyte Membrane/Porous Nanoparticles Loading Mir-137 for NIR-Stimulated Theranostics of Glioblastomas

**DOI:** 10.3390/nano12091464

**Published:** 2022-04-26

**Authors:** Minghui Li, Xinyu Cui, Feng Wei, Chao Li, Xiaojun Han

**Affiliations:** 1 State Key Laboratory of Urban Water Resource and Environment, School of Chemistry and Chemical Engineering, Harbin Institute of Technology, Harbin 150001, China; lmh17351@163.com (M.L.); 2008dadapang@163.com (F.W.); 17b925070@stu.hit.edu.cn (C.L.); 2 Department of Pharmaceutics, Daqing Campus of Harbin Medical University, Daqing 163319, China; 3 Department of Public Health, Mudanjiang Medical University, Mudanjiang 157000, China; xinyucui@outlook.com

**Keywords:** mesoporous silica, indocyanine green, microRNAs-137, red blood cell membranes, glioblastomas, synergistic treatment

## Abstract

Cell-derived drug carriers have increasingly gained the interest of the scientific community due to their ability to imitate various natural properties of their source cells. We developed theranostics nanoplatforms composed of mesoporous silica nanoparticles (MSNs), indocyanine green (ICG) molecules, microRNAs-137 (miR-137), red-blood-cell membranes (RM), and tumor-targeting cyclo Arg-Gly-Asp-d-Phe-Cys peptides (cRGD(fC)), which were abbreviated as MSNs/ICG/miR/RM/RGD particles. These particles possessed photothermal and gene therapy properties due to ICG and miR-137, respectively. The photothermal conversion efficiency was ~18.7%. Upon 808 nm light irradiation, the tumor inhibition rate reached 94.9% with dosage of 10 mg/kg. The developed nanoplatform possessed unique properties, such as exceptional biocompatibility, immune escaping, and specific recognition, which was also used for near-infrared fluorescence, photoacoustic (PA) bimodal imaging-guided tumor recognition.

## 1. Introduction

Glioblastoma (GBM) is the most aggressive malignant type of astrocytoma, responsible for millions of deaths worldwide [1,2]. The median survival of GBM patients remains less than two years [3,4]. To improve the therapeutic success rate, immunotherapy, gene therapy (GT), photodynamic therapy (PDT), and photothermal therapy (PTT) were developed [5,6,7,8].

PTT, a new noninvasive oncology tumor therapy with high selectivity and low side effects, has attracted the attention of scientists [9,10]. It utilizes the photothermal effect to increase the temperature of the surrounding environment and trigger the death of cancer cells. The photothermal agents are classified into inorganic and organic agents. The vast majority of inorganic photothermal agents (such as noble metal, semiconductor, and carbon-based nanomaterials, etc.) [11,12,13,14] were hindered by unknown biological toxicity, non-degradability, and rapid clearance, resulting in unsatisfactory efficiency. In this scenario, near-infrared absorption organic materials were considered as the most promising candidates, owing to their superior biocompatibility, potential biodegradability, and high reproducibility. Indocyanine green (ICG), a photothermal agent approved by the U.S. Food and Drug Administration (FDA) [15,16], with negligible toxicity, excellent photostability, and injectability, has been a promising candidate for photothermal therapy. Notably, the emission and high photothermal conversion ability of ICG at 835 nm exhibit great potential as infrared thermal, near-infrared fluorescence, PA-imaging, contrast agents. It was used as an indicator to track the movement of nanomaterials in real-time [17].

Gene therapy (GT), which employs nucleic acid as a medication to treat a variety of ailments, has grown increasingly popular [18,19]. Since microRNAs modulate post-transcriptional gene regulation, microRNA mimics or antagomirs were used to correct specific microRNA aberrations to treat tumors. In GBM, microRNA-137 (miR-137) serves as a tumor suppressor, inhibiting cancer cells from proliferating and invading [20,21]. However, the miRNA carriers had limitations, such as low efficiency of cellular uptake, poor specificity of miRNA release to cytoplasm, and rapid biodegradation. Therefore, it is essential to develop the efficient nanocarriers to deliver genes into target cells.

Red cell membrane-based drug delivery systems have drawn great attention [22]. The surface of erythrocyte membranes contains a variety of proteins (such as membrane protein, CD47 protein, and polysaccharides), which endow nanoparticles with longer circulation time, less reticuloendothelial system (RES) uptake chance, and less immune recognition. Therefore, the cell-membrane-engineering strategy has been widely used in inorganic nanomaterials, such as Fe_3_O_4_ nanoparticles, gold nanoparticles, Prussian blue particles, and silica nanoparticles [22,23,24,25]. However, it is still a great challenge to enhance the active targeting and prolong circulating time. The RGD peptide was used to modify the surface of the erythrocyte membrane, which selectively binded to α_V_β_3_ and α_V_β_5_ integrins overexpressed in tumor neovasculature and glioma cells [26,27], could facilitate their transcellular transport into a GBM model. Thus, RGD peptides implanted in erythrocyte membranes in combination with potent GBM cell medicines could increase the treatment efficacy.

**Scheme 1:**
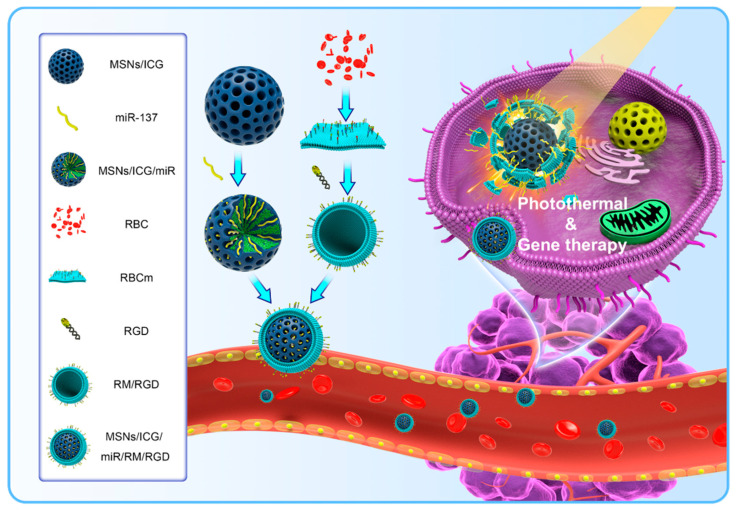
Schematic illustration of MSNs/ICG/miR/RM/RGD particles for tumor treatment and bio-imaging.

Herein, we devised NIR-responsive MSNs/ICG/miR/RM/RGD particles for PTT/GT synergistic tumor therapy (Scheme 1). The red cell membrane (RM) was camouflaged on the surface of MSNs/ICG/miR particles, and responsible for long circulation and immune escape. To obtain superior tumor accumulation and therapeutic efficacy, cRGD(fC) peptides were introduced on the surface as targeting molecules. The prepared nanoparticles were used to bio-image and treat GBM in nude mice.

## 2. Materials and Methods

### 2.1. Reagents

MicroRNA-137 mimics (mature microRNA sequence: 5′-UUAUUGCUUAAGAAUACGCGUAG-3′) were obtained from GenePharma Co., Ltd. (Shanghai, China). The 1, 2-distearoyl-sn-glycero-3-phos-phoethanolamine-N-[maleimide (polyethyleneglycol)] (DSPE-PEG2000-MAL, 2.0 kDa) was obtained from NOF Corporation (Tokyo, Japan). The cRGD(fC) peptide was synthesized by Top-Peptide Co., Ltd. (Shanghai, China). Hoechst33342, PI, and calcein-AM were purchased from Shanghai solarbio Bioscience & Technology Co., Ltd. (Shanghai, China). Cetyltrimethylammonium bromide (CTAC) and FITC were purchased from Sigma-Aldrich. The 3-aminopropyltriethoxysilane (APTES), tetraethyl orthosilicate (TEOS), ethylene imine polymer (PEI, M.W. 600), and indocyanine Green (ICG) were obtained from Innochem Technology Co., Ltd. (Beijing, China). The penicillin−streptomycin solution and fetal bovine serum (FBS) were bought from Zhejiang Tianhang Biotechnology Co., Ltd. (Hangzhou, China).

### 2.2. Characterizations of MSNs/ICG/miR/RM/RGD

A Zetasizer Nano-ZS90 (Malvern, UK) was used to measure the size and zeta potential of samples. Scanning electron microscopy (SEM, Quanta 200 FEG, Portland, OR, USA), transmission electron microscopy (TEM, Tecnai-F30, Portland, OR, USA), and atomic force microscope (AFM) were used to study the morphology of samples. The drug release rate and loading efficiency were obtained using fluorescence spectrophotometer (PerkinElmer, Waltham, MA, USA). X-ray powder diffraction (XRD) measurements were recorded by D2 PHASER Bruker AXS GmbH (Karlsruhe, Baden-Württemberg, Germany).

### 2.3. Fabrication of MSNs/ICG

A total of 10 mg ICG and 3 mg PEI were dissolved in 2 mL DMSO solution, which was mixed with 24 mL CTAC and 0.2 g triethylamine in deionized water at 60 °C for 1 h. Then, 4 mL TEOS and 16 mL cyclohexane were added to the above mixed solution slowly, followed by stirring at 60 °C for 12 h. The product was rinsed at 60 °C with ammonium nitrate ethanol solution several times to obtain ICG doped MSNs.

To prepare FITC labelled MSNs (MSNs-FITC), 15 mg FITC and 100 µL APTES were dissolved in 5 mL anhydrous ethanol with stirring for 24 h, followed by adding 2 mL FITC-APTES into 30 mg dispersed MSNs at room temperature and stirring overnight. The product was centrifuged at 7000 rpm for 10 min and washed with deionized water for three times.

A total of 10 mg MSNs/ICG was mixed with 5 mL DEPC solution containing 4.95 μg Cy3 labeled miR-137 overnight, followed by the removal of the unloaded Cy3 labeled miR-137 by centrifuging at 7000 rpm for 10 min to obtain the MSNs/ICG/miR particles. The mass of unloaded Cy3 labeled miR-137 in the suspension was 0.45 μg.

### 2.4. Preparation of Red Blood Cell Membrane Vesicles

The red blood cells (RBC) were extracted from BALB/c nude mice. The RBC were freshly separated by diluting mouse blood with 1 × PBS followed by centrifuging at 800× *g* for 5 min at 4 °C. The purified RBC ghost was suspended in 0.2 × PBS for 30 min at 4 °C and centrifuged at 10,000× *g* for 30 min at 4 °C for three times. To obtain red blood cell membrane vesicles (RVs), the 50 μL RBC ghost was diluted to 1 mL PBS and then extruded through 0.4 and 0.1 μm polycarbonate membranes 21 times, respectively.

### 2.5. Preparation of MSNs/ICG/miR/RM/RGD Particles

A total of 10 mg MSNs/ICG was mixed with 5 mL DEPC solution containing miR-137 (0.15 OD) overnight, followed by removing unloaded miR-137 by centrifuging at 7000 rpm for 10 min three times to obtain the MSNs/ICG/miR particles. The as-prepared RVs at the size of 100 nm was mixed with MSNs/ICG/miR at the mass ratio of 1:2 and in an ultrasonic bath for 5 min, leading to the RM reconstruction on the surface of MSNs. The thiol group of cRGD(fC) polypeptide was conjugated with maleimide of DSPE-PEG-MAL to produce DSPE-PEG-RGD. A total of 9 mg of DSPE-PEG-MAL (MW: 2890) and 2 mg of cRGD (fC) peptides (MW: 578.66) were respectively dissolved in 1 mL of buffer solution (pH 7.4) and then gently mixed at 4 °C for 12 h. Then, the DSPE-PEG-RGD conjugates were purified by dialysis. The product was detected by ^1^H NMR spectrum. The results demonstrated that the peak disappeared at 6.3 ppm in the ^1^H NMR spectra of DSPE-PEG-RGD, indicating that cRGD(fC) was successfully conjugated to DSPE-PEG-MAL (Figure S1). Then, 200 μL DSPE-PEG-RGD was incubated with 2 mL MSNs/ICG/miR/RM to obtain MSNs/ICG /miR/RM/RGD. A 10 min centrifugation at 6000 rpm at 4 °C was performed to remove the possible excess DSPE-PEG-RGD in supernatant. The collected RGD modified MSNs/ICG/miR/RM were then suspended in 2 mL PBS. 

### 2.6. Photothermal Effect

A sample solution (PBS, MSNs/ICG/RM, or MSNs/ICG/RM/RGD, 2 mg/mL) was added in a cuvette and irradiated with the 808 nm laser (0.5 W/cm^2^). The temperature was recorded every 30 s with an infrared thermal image camera.

### 2.7. Electrophoretic Analysis of Sodium Dodecyl Sulfate Polyacrylamide Gel (SDS-PAGE)

The proteins of MSNs/RM were characterized by SDS-PAGE. Membrane proteins from RM, RVs, or MSNs/ICG/RM were analyzed by the bicinchoninic acid (BCA) assay kit (Beyotime Biotechnology, Shanghai, China). All samples were mixed with loading buffer at a 3:1 volume ratio and heated to 100 °C for 5 min. The same amount of protein sample was added into the well of 12% SDS-PAGE. The SDS-PAGE buffer was used as the running buffer. The gel was stained with coomassie bright blue.

### 2.8. Cell Culture

U87 glioma cells and mouse macrophage RAW264.7 cells were obtained from Cell Bank of Shanghai, Chinese Academy of Sciences (Shanghai, China). The U87 cells were cultured in high-glucose Dulbecco’s modified Eagle medium (DMEM) containing 10% (*v*/*v*) FBS and 1% (*v*/*v*) antibiotics (penicillin−streptomycin, 100 U/mL). The RPMI 1640 medium was chosen for the maintenance of the RAW264.7 cells. Both abovementioned cells were incubated at 37 °C in 95% air and 5% CO_2_ incubator.

### 2.9. In Vitro Release

The MSNs/ICG/miR/RM/RGD (miR-137 labeled by Cy3) were prepared using the abovementioned method. The supernatant of free Cy3-labeled miR-137 was collected and used to calculate the loading efficiency. The sample (0.5 mL) was added into the bag filter (MW: 8000) with 20 mL PBS as the release medium and stirred on the magnetic-stirring apparatus with the same stirring speed. The release medium (2 mL) was collected at different times, and the same volume of the fresh release medium was added back. The collected samples were examined by fluorescence spectrometer at 552 nm.

### 2.10. In Vitro Cellular Uptake

The U87 and RAW264.7 cellular uptake were examined by using a confocal laser scanning microscope (CLSM, Olympus, FV 3000, Tokyo, Japan). The U87 cells or RAW264.7 cells were seeded in a confocal Petri dish (Nest, Wuxi, China) for 12 h. The FITC-labeled MSNs, MSNs/RM, or MSNs/RM/RGD (MSNs, 2 mg/mL) were uniformly dispersed in the DMEM or RPMI complete medium at 37 °C for 4 h in the culture medium. The cells were washed by PBS three times, then stained with Hoechst33342 solution for 5 min to stain the nuclei. The cellular uptake was observed by CLSM.

### 2.11. Cytotoxicity In Vitro

The in vitro cytotoxicity of MSNs/ICG/RM/RGD was detected by hemolysis and CCK-8 assay, respectively. Blood was centrifuged at 800× *g* for 5 min and washed three times with 1 × PBS to obtain pure RBC. Afterward, RBC (25 μL) were mixed with 1.0 mL MSNs/ICG/RM/RGD suspension with a series of concentrations for 8 h. MSNs/ICG/RM/RGD were dissolved in deionized water as a positive control and in PBS as a negative control, respectively. After centrifugation, the absorption values of supernatant were measured.

The U87 cells were seeded into 96-well plates at a density of 1 × 10^3^ cells per well. After 12 h of culture, the cells were treated with a series of concentrations of MSNs/ICG/RM/RGD nanoparticles (0, 25, 50, 100, and 250 μg/mL) for 24 h or 48 h. Then, the CCK-8 solution was added to each well to measure its absorption strength at 450 nm to calculate cell viability.

### 2.12. In Vitro Gene/Phototoxicity

The U87 cells were cultured in 6-well microplates (1 × 10^5^ cells per well) for 24 h. Then, the cells were added to the medium with group I: PBS, group II: MSNs/ICG/miR/RM/RGD, group III: MSNs/ICG/miR/RM + NIR, group IV: MSNs/ICG/RM/RGD + NIR, and group V: MSNs/ICG/miR/RM/RGD (MSNs/ICG: 2 mg/mL) for 4 h. For PTT, groups III, IV, and V were irradiated with an 808 nm NIR laser (0.5 W/cm^2^, 10 min). The culture media were replaced with fresh media to culture for a further 24 h. After carefully rinsing, the PBS containing calcein-AM (for living cells) and PI (for dead cells) were added to stain the cells for 10 min. The cells were washed with PBS and observed by CLSM.

### 2.13. Quantification of Real-Time PCR Study

For the detection of miR-137, the U87 cells were seeded at 5 × 10^4^ cells per well into 6-well plates and maintained in culture with MSNs/ICG/miR, MSNs/ICG/miR/RM, MSNs/ICG/miR/RM/RGD, or PBS. After incubation of 48 h, the fresh culture medium was replaced. Real-time PCR was performed according to studies in Roch Real-Time PCR System. Total RNA was extracted using TRIzol reagent. The RNA levels of samples were quantified using AceQ qPCR SYBR Green Master Mix and were normalized to the levels of β-actin expression.

### 2.14. Tumor Model Establishment

BALB/c nude mice (4–5 weeks old, male and female in half) were used for the animal experiment after purchasing them from Beijing Vital River Laboratory Animal Technology Co., Ltd. and maintained under SPF condition.

To establish the tumor model, BALB/c nude mice were subcutaneously implanted with 2 × 10^7^ U87 cells into the left back legs. The tumor volume reached approximately 100 mm^3^ before treating with drug-loaded particles. The tumor volume was recorded by the formula: V = L × W^2^/2 (L, longest dimension; W, shortest dimension).

### 2.15. Infrared Thermal Imaging

The U87 tumor-bearing mice were randomly divided into three groups (*n* = 5) by intravenous injection of PBS, MSNs/ICG/RM and MSNs/ICG/RM/RGD (200 μL, 2 mg/mL). After 12 h, the mice were anesthetized and irradiated with an 808 nm laser (1.0 W/cm^2^) for 10 min at the tumor site. An infrared thermography was utilized to monitor temperature changes.

### 2.16. NIR-Fluorescence Imaging In Vivo

Due to the strong fluorescence emission in the NIR region, the biological distribution of MSNs/ICG/RM/RGD can be analyzed by fluorescence imaging. When the tumor volume reached ca. 100 mm^3^, the tumor-bearing mice were randomly divided into two groups. The mice were injected with MSNs/ICG/RM or MSNs/ICG/RM/RGD (200 μL, 2 mg/mL) via tail veins. The fluorescence signal of ICG was obtained by GRAND-imaging system (Wuhan, China) (λex: 704 nm; λem: 735 nm).

### 2.17. In Vitro and In Vivo PA Imaging

The MSNs/ICG/RM/RGD were dispersed in PBS of 0, 10, 25, 50, and 100 μg/mL at diverse concentrations and then added into PU tubes for in vitro PA imaging by virtue of photoacoustic microscope. The relevant images of tumor-bearing mice were collected by photoacoustic instruments at 0, 5, 30, and 60 min after intratumorally injecting MSNs/ICG/RM/RGD (2 mg/mL).

### 2.18. In Vivo Antitumor Effect and Biosafety Test

After 10 days of implantation with the tumor size of approximately 100 mm^3^, the tumor-bearing mice were randomly divided into five groups (*n* = 5) and treated with group I (PBS as the control), group II (MSNs/ICG/miR/RM/RGD), group III (MSNs/ICG/miR/RM + NIR), group IV (MSNs/ICG/RM/RGD + NIR), and group V (MSNs/ICG/miR/RM/RGD + NIR) via intravenous injection (10 mg/kg), respectively. At 12 h after injection, the tumor region was irradiated by an 808 nm laser at power density of 1 W/cm^2^ for 10 min.

Weight and tumor size were recorded every two days and normalized in contrast with their initial values. After 15 days, the mice were sacrificed to collect the main organs (heart, liver, spleen, lung, and kidney) and tumor tissues, which were fixed with 4% formaldehyde, embedded in paraffin, and stained with hematoxylin and eosin (H&E) and Ki67 to observe cell apoptosis and necrosis or the late proliferation of the tumors using the histochemical method.

## 3. Results and Discussion

### 3.1. Preparation and Characterization of MSNs/ICG/miR/RM/RGD

**Figure 1:**
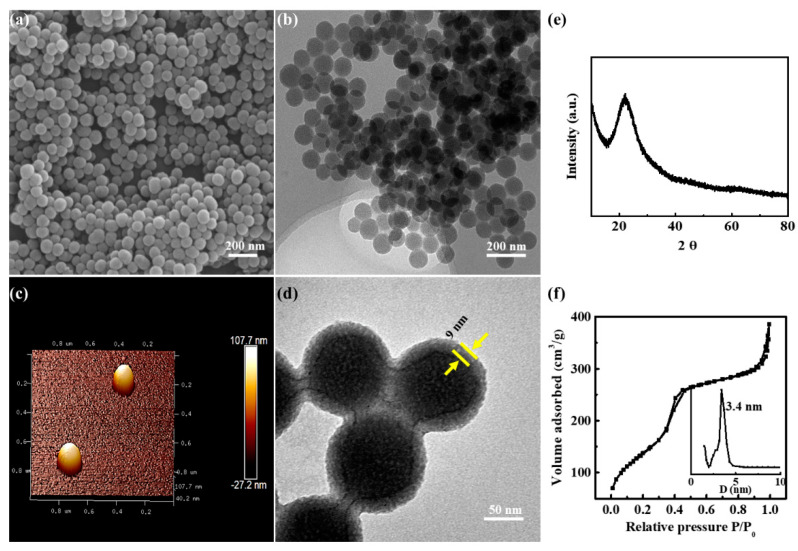
Characterization of samples. (**a**) Representative SEM image of MSNs/ICG. (**b**) Representative TEM image of MSNs/ICG. (**c**) AFM image of MSNs/ICG. (**d**) TEM image of MSNs/ICG/RM/RGD. (**e**) XRD analysis of MSNs/ICG. (**f**) BET analysis of MSNs/ICG.

The SEM, TEM, and AFM images (Figure 1a–c) showed that MSNs/ICG particles were uniform, well-dispersed spheres with an average size of ~90 nm. In Figure 1e, the XRD pattern of MSNs/ICG exhibited a broad diffraction peak at ca. 22°, assigning to the amorphous silica framework. The nitrogen adsorption–desorption isotherm and pore size distribution curves of MSNs/ICG (Figure 1f) exhibited typical IV isotherm with a narrow pore size distribution (3.4 nm), demonstrating the existence of a mesoporous structure. BET surface area and pore volume of MSNs/ICG were determined to be 773.1 m^2^/g and 0.95 mL/g, respectively, indicating high-loading capacity of the drug. The TEM image (Figure 1d) of MSNs/ICG/RM/RGD displayed a typical core-shell structure with an outer RM/RGD layer thickness of ~9 nm. For reflecting actual dimension of MSNs/ICG/RM/RGD in PBS, the hydrodynamic diameters of MSNs/ICG/RM/RGD were monitored by dynamic-light-scattering (DLS) measurement and determined to be 107.4 ± 2.3 nm (Figure S2a), which was a suitable size for further in vivo administration. The zeta potential also confirmed that the modification of the MSNs/ICG surface with the RM/RGD was successful (Figures S2b and S3a). Notably, the obtained MSNs/ICG/RM/RGD were relatively stable with only a slight increase in particle size in PBS or 10% FBS (Figure S3b). Furthermore, the miR-137 (Cy3-labeled) loading efficiency was 0.45 μg miRNA/1 mg nanoparticles. The in vitro release profile of miR-137 (Cy3-labeled) from MSNs/ICG/miR/RM/RGD was presented in Figure S3c. MSNs/ICG/miR exhibited a sudden miR-137 release profile in PBS with 83.2% cumulative release within 24 h. After RM/RGD camouflaging, MSNs/ICG/miR/RM/RGD illustrated a well stability with less release (16.2%) over the same time. More importantly, the complex membrane proteins on RM played a vital role in escaping the immune system. The membrane proteins in RM, RVs, and MSNs/ICG/RM particles were detected by SDS-PAGE (Figure S4a). The protein bands of MSNs/ICG/RM particles were similar to those of RM and RVs (*n* = 3). The FT–IR spectrum of RM/RGD contained both the characteristic peaks of RM and RGD (Figure S4b), implying that RGD was successfully incorporated into the erythrocyte membrane.

**Figure 2:**
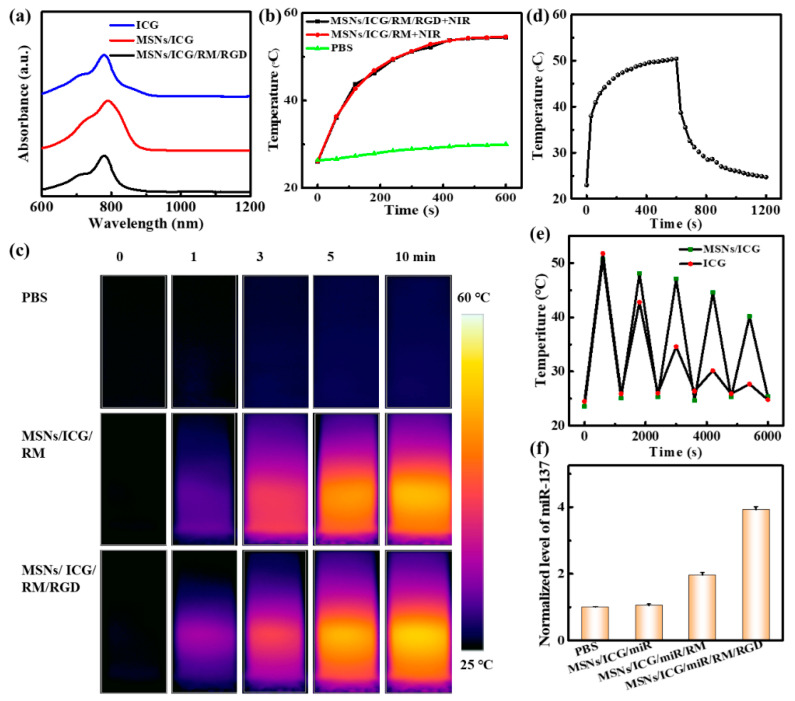
Optical, photothermal properties of samples. (**a**) The UV–Vis–NIR absorption spectra of samples. (**b**,**c**) Temperature profile and infrared thermal images under 808 nm laser irradiation (0.5 W/cm^2^). (**d**) Temperature change of MSNs/ICG aqueous dispersion for 10 min laser irradiation, followed by cooling. (**e**) Temperature curves of MSNs/ICG and free ICG with repeated irradiation on/off cycles under 808 nm laser irradiation. (**f**) The normalized miR-137 level in various nanoparticles quantified by qRT-PCR.

The optical absorption in the NIR region was the basic requirement for the MSNs/ICG/RM/RGD to implement PTT. MSNs/ICG/RM/RGD showed a distinctive absorbance peak at 780 nm, indicating the successful ICG doping (Figure 2a). For an investigation of the NIR laser-induced photothermal effect, MSNs/ICG/RM/RGD, MSNs/ICG/RM, and PBS were irradiated by an 808 nm laser (0.5 W/cm^2^, 10 min), and the corresponding temperature changes were recorded by infrared thermography. As depicted by Figure 2b,c, the temperatures of MSNs/ICG/RM and MSNs/ICG/RM/RGD all reached ~53 °C. In contrast, the temperature of PBS just increased from room temperature to ~27.2 °C, thus proving that MSNs/ICG/RM/RGD possess excellent photothermal properties. On conducting a temperature-cooling temperature test on MSNs/ICG/RM/RGD and linear fitting of cooling time (Figure 2d and Figure S4c), the photothermal conversion efficiency of MSNs/ICG/RM/RGD was calculated to be ~18.7%, according to previously reported methods [28]. It is noteworthy that the stability of MSNs/ICG/RM/RGD was significantly higher than that of free ICG after five consecutive cycles of irradiation (Figure 2e).

### 3.2. Biocompatibility, Immune Evading, and Targeting Ability of Nanoparticles

**Figure 3:**
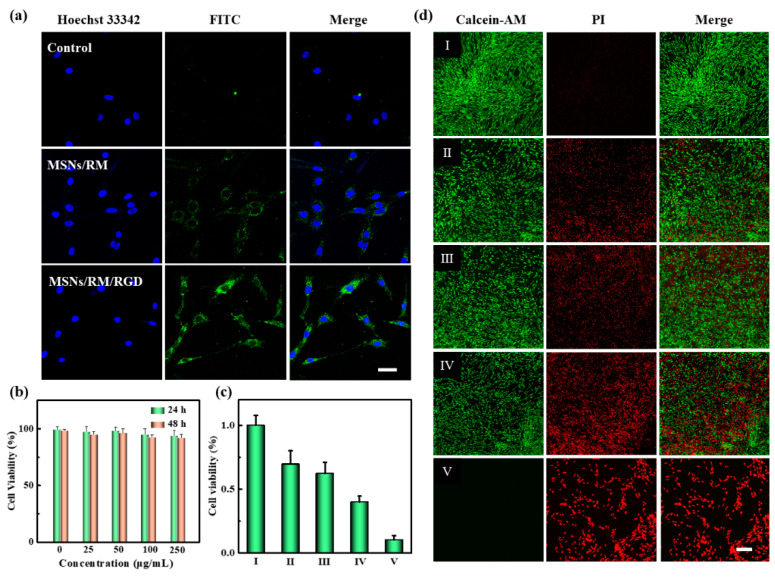
Targeting ability, biocompatibility, and cytotoxicity of nanoparticles in vitro. (**a**) CLSM images of U87 cells after incubating with MSNs, MSNs/RM, and MSNs/RM/RGD (MSNs labeled with FITC) for 4 h. Scale bar = 20 μm. (**b**) The cell viabilities treated with MSNs/RM/RGD with different concentrations. (**c**) The cytotoxicity of (**d**) live/dead staining of U87 cells in different groups. (I) Control, (II) MSNs/ICG/miR/RM/RGD, (III) MSNs/ICG/miR/RM + NIR, (IV) MSNs/ICG/ RM/RGD + NIR, and (V) MSNs/ICG/miR/RM/RGD + NIR. Scale bar = 100 μm.

To verify the biocompatibility of MSNs/ICG/RM/RGD particles, the hemolysis assay and CCK-8 assay were performed, respectively. As depicted in Figure S5, there was no conspicuous hemolysis when varied doses of MSNs/ICG/RM/RGD particles were added, and the associated blood absorption was evaluated. The low hemolysis rate was less than 5% even in a concentration of 250 μg/mL. The CCK-8 results in the U87 cells showed that the cell viability was more than 90% with the same high concentration (Figure 3b). In addition, RM-coated nanoparticles also possessed immunological safety [29].

The ability to evade the immune system of cell-engineering nanoparticles was examined by anti-phagocytosis against RAW264.7 cells. To track nanoparticles, MSNs were labeled with a green fluorescent dye FITC. As shown in Figure S6, in the sample of RAW264.7 cells incubated with MSNs/RM and MSNs/RM/RGD, a visible faint green fluorescence was seen compared to cells incubated with MSNs, indicating that the phagocytosis of MSNs was significantly decreased after RM coating.

With excellent biocompatibility and immune escape capability, we subsequently evaluated the tumor-targeting capability and therapeutic efficacy of the targeted cRGD(fC)-modified formulation. The U87 cells treating with MSNs/ICG/RM/RGD exhibited the highest fluorescence intensity in contrast to non-targeted ones, whereas the MSN sets showed the least fluorescence intensity (Figure 3a). This result implied that RGD-modified erythrocyte membrane carriers could be used for the targeted delivery of miRNAs into cells and achieve endosomal escape [30].

### 3.3. Antitumor Activity In Vitro

The in vitro viability of the U87 cells treated with MSNs/ICG/RM/RGD were evaluated using CCK-8 assay coupling with live/dead staining. In Figure 3c, the MSNs/ICG/miR/RM/RGD, MSNs/ICG/miR/RM + NIR, and MSNs/ICG/RM/RGD + NIR groups showed insufficient cytotoxic activity against the U87 cells (69.5%, 62.1%, and 39.8% for cell survival rates, respectively). In contrast, only 10% of cells survived when the cells were treated with MSNs/ICG/miR/RM/RGD + NIR. Furthermore, PI and calcein-AM were employed to stain cells to distinguish between the dead and live cells in different groups. As depicted in Figure 3d, the cell treated with MSNs/ICG/miR/RM/RGD + NIR exhibited the stronger anti-proliferative effect than those treated with MSNs/ICG/miR/RM + NIR, owing to its active targeting capability and increasing cellular uptake. In contrast, comparing the groups of MSNs/ICG/miR/RM/RGD and MSNs/ICG/RM + NIR, the group of MSNs/ICG/miR/RM/RGD + NIR exhibited an obvious synergetic antitumor effect of PTT and GT.

The miR-137 was a brain-rich miRNA to be downregulated in gliomas. It could suppress migration and invasion in glioma cell lines and was considered as a tumor suppressor miRNA [31]. The levels of miR-137 regulated by various nanoparticles in the U87 cell line were detected by quantitative real-time polymerase chain reaction (qRT-PCR). The results revealed that the U87 cells treating with MSNs/ICG/miR/RM and MSNs/ICG/miR/RM/RGD significantly increased miR-137 level by 1.96 and 3.92-fold, respectively (Figure 2f), which confirmed the effective delivery of miR-137 by MSNs/ICG/miR/RM/RGD.

### 3.4. In Vivo Imaging

**Figure 4:**
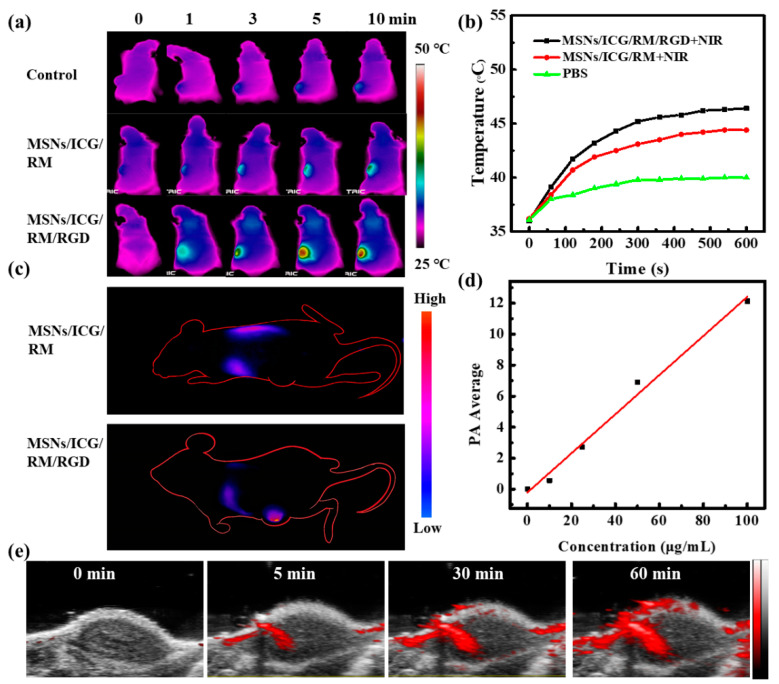
Multifunctional imaging of mice bearing U87 tumors. (**a**) Infrared thermal images of U87 tumor-bearing mice at 0, 1, 3, 5, and 10 min after tail vein injection of PBS as control, MSNs/ICG/RM, or MSNs/ICG/RM/RGD under laser exposure (808 nm, 1 W/cm^2^). (**b**) Temperature changes in the tumor area at different time points after laser treatment. (**c**) In vivo NIR-fluorescence images of U87 tumor-bearing mice. (**d**) The linear relationship between the PA signal intensity and the concentration of MSNs/ICG/miR/RM/RGD. (**e**) PA images of tumor areas at indicated time points (0, 5, 30, 60 min) after intratumoral injection of MSNs/ICG/miR/RM/RGD.

To demonstrate the MSNs/ICG/RM/RGD imaging functionality, a multi-imaging function study was carried out on a mice model. Firstly, the MSNs/ICG/RM/RGD exhibited strong infrared thermal-imaging signals and significant temperature elevation on the tumor site upon laser irradiation after intravenous injection. On analyzing in vivo photothermal imaging (Figure 4a) and corresponding temperature changes (Figure 4b) under the same irradiation conditions, the mice treated with MSNs/ICG/RM/RGD showed the most notable temperature rise (~13 °C) in comparison with those treated with MSNs/ICG/RM (~9 °C). Given that mice injected with PBS only experienced a ~2 °C temperature shift, MSNs/ICG/RM/RGD could efficiently convert the 808 nm laser into thermal energy. Moreover, benefiting to the strong fluorescence emission of ICG in the NIR region, the in vivo biodistribution of MSNs/ICG/RM/RGD was investigated in the U87 tumor bearing mice. In detail, the U87-tumor-bearing mice were treated with MSNs/ICG/RM and MSNs/ICG/RM/RGD, respectively. An in vivo laser-imaging device was used to track whole-body image alterations after 12 h of intravenous injections. As shown in Figure 4c, there was no obvious NIR-fluorescence at the tumor location treated with MSNs/ICG/RM. By contrast, MSNs/ICG/RM/RGD-treated mice exhibited noticeable aggregation in tumor domain with strong NIR-fluorescence. This suggested that the RGD modification played a role in improving the active target efficacy. The infrared thermal and NIR-fluorescence images were in fairly good agreement. For deeper imaging, the PA signal intensity of MSNs/ICG/RM/RGD was evaluated due to its superior photothermal ability. As depicted in Figure 4d, the increase of PA signal intensity was approximately linear to the concentration of MSNs/ICG/RM/RGD, suggesting a satisfactory PA-imaging potency in vitro. The in vivo PA imaging was performed on the U87-tumor-bearing mice. After the mice were injected with MSNs/ICG/miR/RM/RGD at various time points, the PA-imaging value gradually enhanced (Figure 4e). These findings validate its potential as a PA agent for cancer diagnosis.

### 3.5. Antitumor Efficacy In Vivo

**Figure 5:**
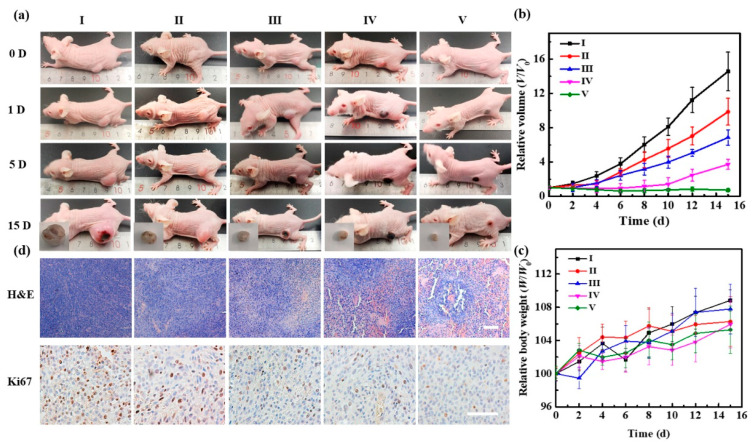
Evaluation of in vivo antitumor efficacy after 15 days of treatments with (I) control, (II) MSNs/ICG/miR/RM/RGD, (III) MSNs/ICG/RM/RGD + NIR, (IV) MSNs/ICG/miR/RM + NIR, and (V) MSNs/ ICG/miR/RM/RGD + NIR. (**a**) Representative images of tumor-bearing nude mice treated with different groups as a function of time. (**b**) Relative volume curve of tumors of different mice groups 15 days after treatment. (**c**) The body weights of difference mice groups 15 days after treatment. (**d**) Representative H&E and Ki67-staining images of the tumors of different groups. Scale bar = 100 μm.

Encouraged by the excellent active-targeting ability of MSNs/ICG/RM/RGD, we further assessed their antitumor efficacy in vivo by monitoring the relative volume changes of tumors of groups I−V with a caliper during the 15 days of treatment (Figure 5b). As shown in Figure 5a, mice from group II (tumor inhibition rate of 32.2%) and group III (tumor inhibition rate of 52.9%) performed a lesser extent of tumor inhibition. In contrast, mice treated with MSNs/ICG/miR/RM/RGD and NIR light exposure (group V) showed dramatic tumor regression over time (tumor inhibition rate of 94.9%). As expected, the tumor treated with MSNs/ICG/miR/RM + NIR (group IV) exhibited a lower tumor inhibition rate of 74.4% compared with the group V due to their unfavorable tumor accumulation. Finally, the in vivo toxicity of MSNs/ICG/miR/RM/RGD was evaluated by body weight, H&E, Ki67 staining, and blood routine analysis. A significant fluctuation in the average body weight of any group of mice was not observed in mice from any group during the 15 days of treatment (Figure 5c). H&E and Ki67 staining of tumor sections further validated the antitumor efficacy of those treatments (Figure 5d). H&E staining of tumor tissues indicated large areas of cell deformation in the MSNs/ICG/miR/RM/RGD + NIR group compared with the other groups, which was consistent with the tumor volume study. Nevertheless, no apparent damages or changes were observed in any major organs, thus corroborating the excellent biocompatibility of MSNs/ICG/miR/RM/RGD in vivo (Figure S7). Similarly, on performing Ki67 staining, the MSNs/ICG/miR/RM/RGD + NIR group revealed the least positive signals (brown) compared with other groups. The proliferation of tumors was suppressed significantly after the U87-tumor-bearing mice were treated with MSNs/ICG/miR/RM/RGD. The results of the serum biochemistry test revealed that hematocrit (HCT), hemoglobin (HGB), mean corpuscular hemoglobin (MCH), mean corpuscular hemoglobin concentration (MCHC), mean corpuscular volume (MCV), platelet (PLT), platelet volume (PCT), and red blood cells (RBC) were all within the normal range after 15 days of treatment (Figure S8).

## 4. Conclusions

The NIR-responsive biomimetic nanoplatforms (MSNs/ICG/miR/RM/RGD) exhibited effective antitumor functions owing to the PTT and GT functions. The MSNs/ICG/ miR/RM/RGD nanoparticles possessed superior monodispersity, prolonged blood circulation, and augmented photothermal responses. On injection of MSNs/ICG/miR/RM/ RGD nanoparticles into the tail vein of the tumor-bearing mice, the tumors could be ablated under 808 laser irradiations. NIR fluorescent MSNs/ICG serves as a guide for tumor detection in vivo, realizing the goals of simultaneous tumor treatment and diagnosis. This strategy encourages further development of biomimetic nanoplatforms for precise diagnosis and therapy of glioma.

## Data Availability

Not applicable.

## Supplementary Materials

The following supporting information can be downloaded at: https://www.mdpi.com/article/10.3390/nano12091464/s1, Figure S1: ^1^H NMR spectra of (a) DSPE-PEG-MAL-and (b) DSPE-PEG-RGD in D_2_O. Figure S2: The particle size (a) and zeta potential (b) of MSNs/ICG. Figure S3: (a) Zeta potential of nanoparticles, (b) the hydrodynamic particle size of nanoparticles in PBS and FBS (10%) as a function of time, (c) the cumulative release curve of miR-137 (labeled by Cy3) in different carriers. Figure S4: (a) SDS-PAGE protein analysis, (b) FT–IR spectra of RM, RGD, and RM/RGD, (c) Linear cooling time versus -Ln(θ). Figure S5: The hemolysis assays of MSNs/ICG/miR/RM/RGD. Figure S6: Confocal microscopy images of RAW264.7 cells incubated with various nanoparticles (MSNs labeled by FITC). Figure S7: H&E staining of lung, liver, spleen, kidney, and heart at the end of the antitumor inhibition test. Figure S8: Blood biochemistry analysis of nude mice in five groups.
