# Supplementary material for: RGD Peptide Modified Erythrocyte Membrane/Porous Nanoparticles Loading Mir-137 for NIR-Stimulated Theranostics of Glioblastomas
Source: Nanomaterials (Basel). 2022 Apr 26;12(9):1464. doi: 10.3390/nano12091464 (PMC9105018; doi:10.3390/nano12091464)
Supplement: Supplementary file 1 [file nanomaterials-12-01464-s001.zip › nanomaterials-1663467-supplementary.pdf]

Supplementary Materials

RGD Peptide Modified Erythrocyte Membrane/Porous Nanoparticles Loading Mir-137 for NIR-Stimulated Theranostics of Glioblastomas

Minghui Li [1,2], Xinyu Cui [3], Feng Wei [1], Chao Li [1] and Xiaojun Han [1,*]

[1] State Key Laboratory of Urban Water Resource and Environment, School of Chemistry and Chemical Engineering. Harbin Institute of Technology. Harbin 150001, China; lmh17351@163.com (M.L.); 2008dadapang@163.com (F.W.); 17b925070@stu.hit.edu.cn (C.L.)
[2] Department of Pharmaceutics, Daqing Campus of Harbin Medical University, Daqing 163319, China
[3] Department of Public Health, Mudanjiang Medical University, Mudanjiang 157000, China; xinyucui@outlook.com
* Correspondence: hanxiaojun@hit.edu.cn

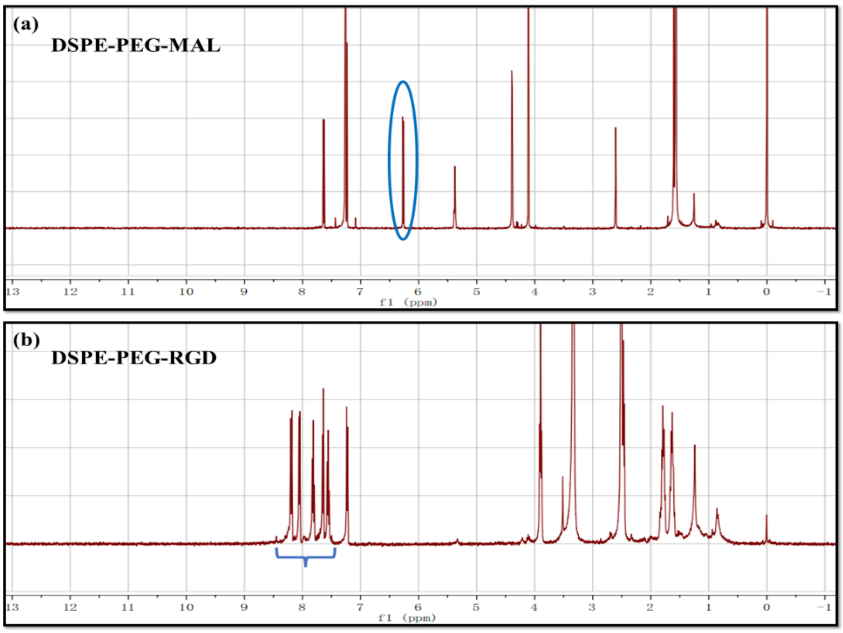

Figure S1. ^{1}H NMR spectra of (a) DSPE-PEG-MAL-and (b) DSPE-PEG-RGD in D_2O.

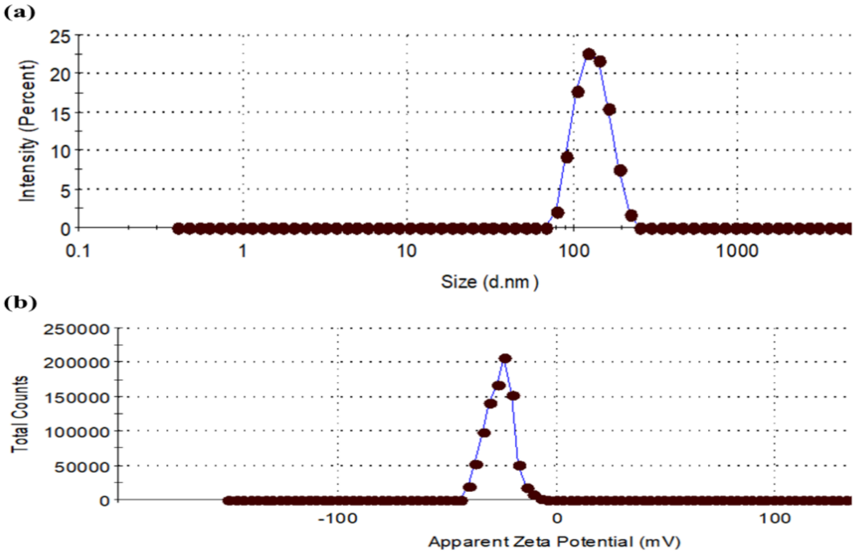

Figure S2. The particle size (a) and zeta potential (b) of MSNs/ICG.

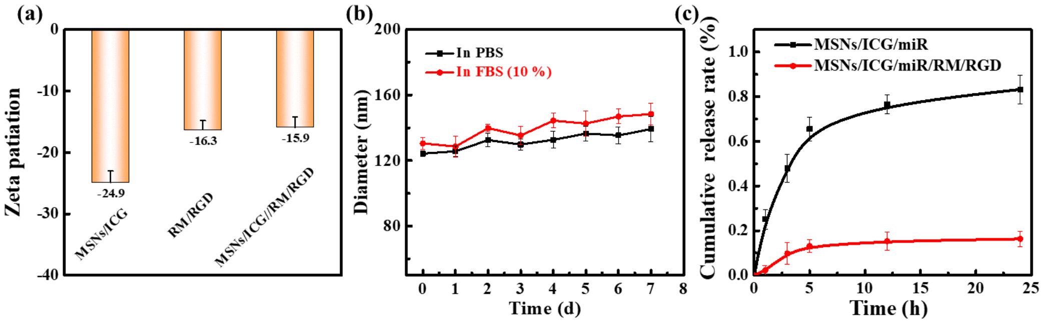

Figure S3. (a)Zeta potential of nanoparticles. (b)The hydrodynamic particle size of nanoparticles in PBS and FBS (10 %) as a function of time. (c) The cumulative release curve of miR-137 (labeled by Cy3) in different carriers.

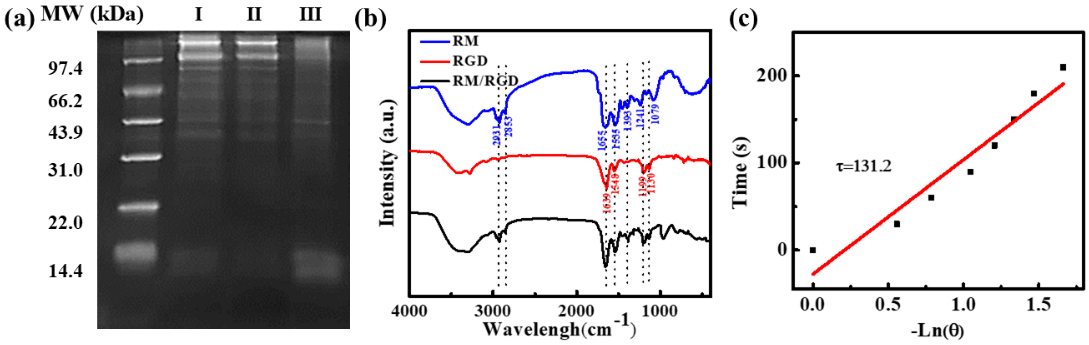

Figure S4. (a) SDS-PAGE protein analysis of (I) RM, (II)RVs, and (III) MSNs/ICG/RM. (b) FT-IR spectra of RM, RGD, and RM/RGD. (c) Linear cooling time versus -Ln(θ).

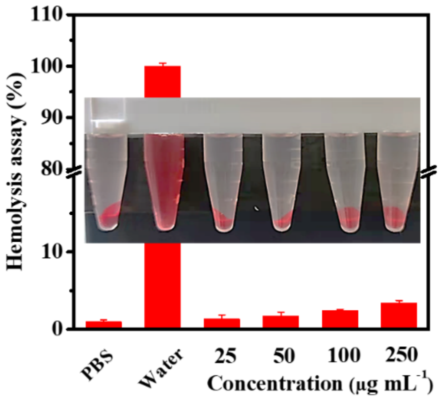

Figure S5. The hemolysis assays of MSNs//ICG/miR/RM/RGD. Digital photos illustrating the hemolysis effects of RBCs after 8 h incubation with MSNs//ICG/miR/RM/RGD in different concentrations. PBS and water were set as negative (−) and positive (+) controls, respectively. Data were presented as mean ± SEM (n = 3).

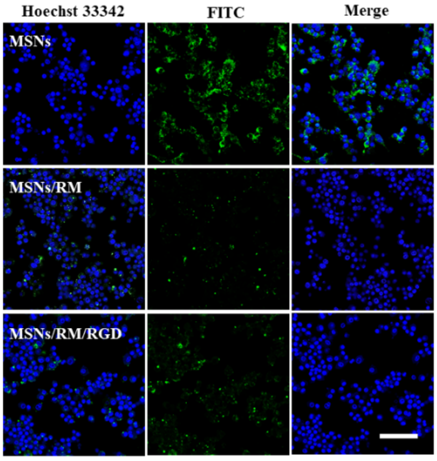

Figure S6. Confocal microscopy images of RAW264.7 cells incubated with various nanoparticles (MSNs labeled by FITC). Scale bar =100 μm.

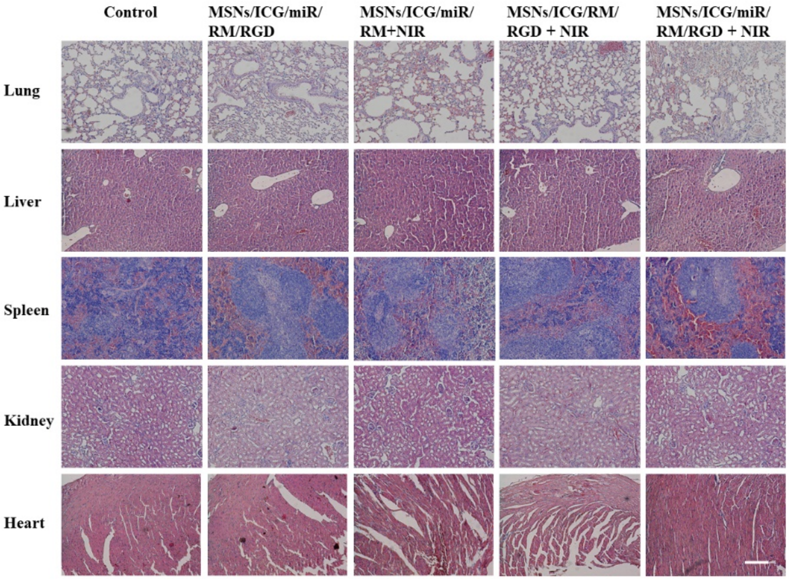

Figure S7. H&E staining of lung, liver, spleen, kidney, and heart, at the end of the antitumor inhibition test. Scale bar =100 μm.

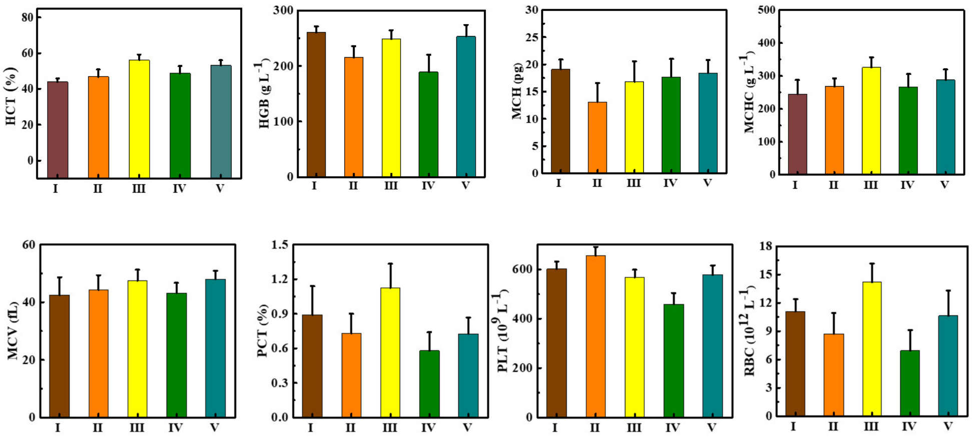

Figure S8. Blood biochemistry analysis of nude mice in five groups. I: PBS, II: MSNs/ICG/miR/RM/RGD, III: MSNs/ICG/miR/RM+NIR, IV: MSNs/ICG/RM/RGD + NIR, and V: MSNs/ICG/miR/RM/RGD + NIR. (n=5).
